# Validation of myocardial perfusion quantification by dynamic CT in an ex-vivo porcine heart model

**DOI:** 10.1007/s10554-017-1171-6

**Published:** 2017-05-23

**Authors:** Gert Jan Pelgrim, Marco Das, Sjoerd van Tuijl, Marly van Assen, Frits W. Prinzen, Marco Stijnen, Matthijs Oudkerk, Joachim E. Wildberger, Rozemarijn Vliegenthart

**Affiliations:** 1 Center for Medical Imaging – North East Netherlands, University Medical Center Groningen, University of Groningen, Hanzeplein 1, P.O. Box EB44, 9713 GZ Groningen, The Netherlands; 2 Department of Radiology and Cardiovascular Research Institute Maastricht (CARIM), Maastricht University Medical Center, Maastricht, The Netherlands; 3 LifeTec Group BV, Eindhoven, The Netherlands; 4 Department of Physiology, Maastricht University, Maastricht, The Netherlands

**Keywords:** Tomography X-ray computed, Myocardial perfusion imaging, Iodine, Ischemia

## Abstract

To test the accuracy of quantification of myocardial perfusion imaging (MPI) using computed tomography (CT) in ex-vivo porcine models. Five isolated porcine hearts were perfused according to Langendorff. Hearts were perfused using retrograde flow through the aorta and blood flow, blood pressure and heart rate were monitored throughout the experiment. An inflatable cuff was placed around the circumflex (Cx) artery to create stenosis grades which were monitored using a pressure wire, analysing perfusion at several fractional flow reserve values of 1.0, 0.7, 0.5, 0.3, and total occlusion. Second-generation dual-source CT was used to acquire dynamic MPI in shuttle mode with 350 mAs/rot at 100 kVp. CT MPI was performed using VPCT myocardium software, calculating myocardial blood flow (MBF, ml/100 ml/min) for segments perfused by Cx artery and non-Cx myocardial segments. Microspheres were successfully infused at three stenosis grades in three of the five hearts. Heart rate ranged from 75 to 134 beats per minute. Arterial blood flow ranged from 0.5 to 1.4 l min and blood pressure ranged from 54 to 107 mmHg. MBF was determined in 400 myocardial segments of which 115 were classified as ‘Cx-territory’. MBF was significantly different between non-Cx and Cx segments at stenosis grades with an FFR ≤0.70 (Mann–Whitney *U* test, p < 0.05). MBF showed a moderate correlation with microsphere MBF for the three individual hearts (Pearson correlation 0.62–0.76, p < 0.01). CT MPI can be used to determine regional differences in myocardial perfusion parameters, based on severity of coronary stenosis. Significant differences in MBF could be measured between non-ischemic and ischemic segments.

**Electronic supplementary material:**

The online version of this article (doi:10.1007/s10554-017-1171-6) contains supplementary material, which is available to authorized users.

## Introduction

Myocardial perfusion imaging (MPI) using computed tomography (CT) has gained interest in recent years, due to new CT scan techniques on state-of-the-art systems. Other non-invasive imaging techniques, like single photon emission computed tomography, magnetic resonance imaging (MRI) and positron emission tomography have proven their value in the detection of myocardial perfusion defects [1]. CT angiography can reliably detect and exclude coronary atherosclerosis [2]. However, CT MPI is still in research phase. Rossi et al. provided an extensive overview of the possibilities of new CT techniques in stress CT MPI analysis [3]. One of the promising CT MPI techniques is quantitative dynamic CT MPI. In dynamic CT MPI, images are acquired at every second heartbeat, resulting in information about the myocardial inflow and outflow of contrast media. Using complex equations, myocardial blood flow (MBF) can be calculated, which provides a quantitative measure for myocardial perfusion [4, 5]. Quantification of MBF will allow monitoring and grading of the severity of ischemia in coronary artery disease and possible detection of global ischemia in multivessel disease, which is often more difficult in non-quantitative imaging [6].

Before clinical implementation of dynamic CT MPI, this technique should be validated in phantom studies and clinical trials. Several studies have already shown promising results in in-vivo animal studies [7–9]. First results of clinical studies showed good diagnostic accuracy [10–15]. Because of the relatively high radiation dose in dynamic CT MPI studies, those studies are more difficult to be performed as an extra study in clinical trials. Ex-vivo studies using animal hearts are an option to validate CT MPI in detail. Blood flow and stenosis grades can be controlled and influenced easily in an ex-vivo setup, allowing for direct manipulation of myocardial perfusion. Using this method, the induction and quantification of myocardial ischemia or infarction can be analysed using dynamic CT MPI techniques. Furthermore, different imaging protocols can be tested in succession, because radiation dose is not an issue in ex-vivo models. The heart of slaughterhouse pigs generally resembles the human heart very well, both in size and in functionality. We have used a CT-compatible porcine heart platform (PhysioHeart^®^, LifeTec Group, Eindhoven, The Netherlands) that allows control of arterial blood flow and coronary stenosis grade. We showed that this Langendorff model can be used in CT without major artefacts [16, 17]. Furthermore, in an ex-vivo model, CT perfusion parameters can be tested against microsphere measurements as a reference standard for absolute MBF In this porcine heart study we aimed to validate the quantification of MBF in dynamic CT MPI at second-generation dual-source CT, compared to FFR and microsphere-derived MBF as reference standards, with the final aim to derive MBF cut-off values for hemodynamically significant CAD.

## Materials and methods

### Heart acquisition and preparation

Ten hearts were obtained from Dutch landrace hybrid pigs (approximately 110 kg) that were slaughtered for human consumption. Protocols at the slaughterhouse and laboratory were in accordance with EC regulations 1069/2009 regarding the use of slaughterhouse animal material for diagnosis and research, supervised by the Dutch Government (Dutch Ministry of Agriculture, Nature and Food Quality), and approved by the associated legal authorities of animal welfare (Food and Consumer Product Safety Authority). Approval of institutional licensing committee for animal experiments was not applicable because the porcine hearts were slaughterhouse waste. For further details about the excision and preparation of the heart the reader is referred to earlier published work [16, 17].

### Platform preparation

**Fig. 1:**
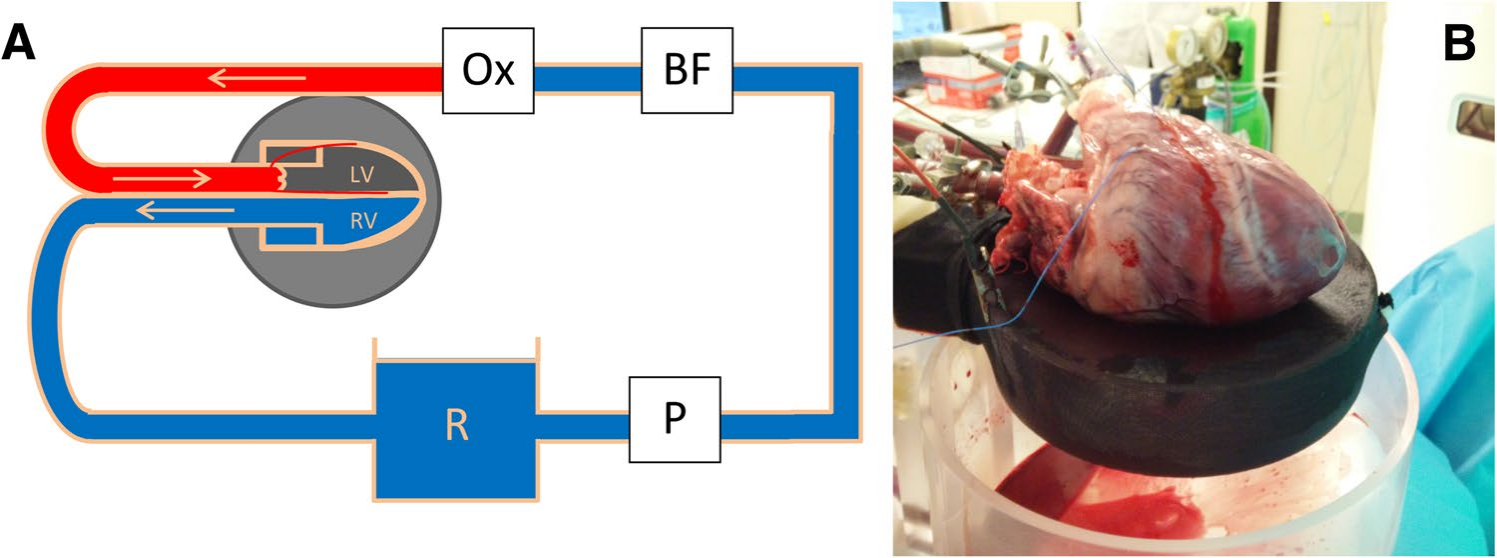
Langendorff perfusion model. **a** Schematic representation of the Langendorff perfusion model. Blood is pumped from the venous reservoir (*R*) through the pump (*P*) to the blood filter (*BF*) after which it is oxygenated using an Oxygenator (*Ox*). The aortic valve is closed due to the pressure applied with the retrograde flow. Due to the closed valve, all blood traverses into the myocardium through the coronaries. Blood leaves the myocardium via the coronary sinus and is pumped from the right ventricle into the venous reservoir. **b** The heart is displayed on a bed of flexible cloth

A period of 2–3 h was required for transport from slaughterhouse to the CT scanner, during which the heart was stored cold. The heart was placed along the z-axis of the scanner on porcine heart platform on flexible clothing to allow free movement of the heart. Placement along the z-axis of the scanner resulted in short-axis images of the porcine hearts. A centrifugal pump (BioMedicus, Medtronic, Minneapolis, Minnesota, USA) circulated the blood in the system. Blood flow was retrograde from the venous reservoir through the aorta to the aortic valve (Fig. 1). Due to the pressure of the retrograde flow, the aortic valve was closed throughout the experiment. The closed aortic valve prevented blood from going into the left ventricle, thereby forcing all the blood to flow through the coronary arteries. The blood was oxygenated with 20% O_2_, 75% N_2_, and 5% CO_2_ gas mixture, using an oxygenator-heat exchanger (AFFINITY^®^ NT Oxygenator; Medtronic, Minneapolis, Minnesota, USA). Blood glucose level was maintained between 5 and 7 mmol/l by addition of glucose-insulin mixture. The temperature of the circulating blood was approximately 38 °C. When reperfusion of the heart was started usually the heart started contracting autonomously. However, to reinstate sinus rhythm some defibrillations were performed using 10–30 J. A Medtronic external pacemaker model 5375 (Medtronic, Minneapolis, Minnesota, USA) was used to stabilize heart rhythm, only in case of unstable heart rhythm. Electrocardiography (ECG) leads were placed on the flexible heart bed of the platform. Wetting the flexible bed with saline provided excellent conduction for ECG measurements. Throughout the entire experiment, arterial blood flow (ABF), mean arterial pressure (MAP), pacing status and heart rate were monitored.

The circumflex (Cx) artery was dissected from the surrounding tissue to allow an inflatable cuff to be placed around the artery, providing the option to induce a proximal stenosis in the Cx. The Cx was selected because of the ease of access and the limited area of perfusion. The induction of the stenosis in Cx territory causes perfusion defects in approximately two myocardial segments per short axis slice. A stenosis in the left anterior descending (LAD) artery would cause larger perfusion defects and would influence the stability of the perfusion experiment. A stenosis in the right coronary artery (RCA) would not show a major perfusion defect in the left ventricle, as the porcine RCA is mainly supplying the right ventricle. The induced stenosis was monitored using a pressure wire, providing information about the pressure drop across the stenosis, fractional flow reserve (FFR). Five stenosis grades were used to study perfusion defects using dynamic myocardial perfusion CT: no stenosis, FFR 0.7, FFR 0.5, FFR 0.3 and occlusion. A standard error of 0.05 for each stenosis grade/FFR value was considered acceptable. The inflatable cuff was released after each perfusion scan to allow reperfusion and recovery of the Cx territory. Therefore, each myocardial perfusion scan was treated as an independent measurement.

### CT protocol

**Fig. 2:**
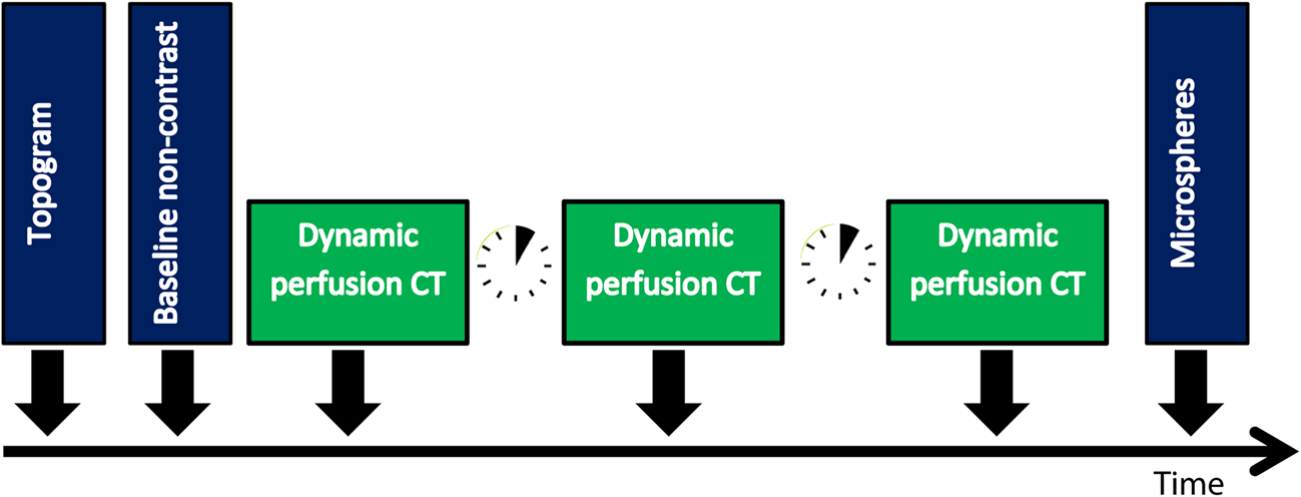
Scan protocol. Scan protocol used for each FFR stenosis grade. Based on the first scan, the scan range is determined. In case nothing changed in the setup, the topogram and non-contrast scans were not repeated for increasing stenosis grades

Second-generation DSCT (SOMATOM Definition Flash, Siemens Healthcare GmbH, Forchheim, Germany) was used to acquire the scans of the porcine hearts. The scan range was determined based on the scout images. One baseline non-contrast enhanced spiral scan with a tube voltage of 100 kV, an effective tube current of 350 mAs with a pitch of 1.2 and a gantry rotation time of 280 ms. This scan was performed to determine background enhancement and scan field of view (FOV). The imaging at each stenosis grade consisted of three dynamic CT perfusion scans. After each contrast-enhanced scan acquisition, a time interval of 5 min was applied to allow contrast concentration in the heart to return to baseline (Fig. 2). Contrast agent was injected 200 cm before the aortic valve to allow baseline measurements without contrast in the inflow tube (>200 cm long). An additional advantage of the long inflow tube was that the blood and contrast were properly mixed before entering the coronaries. In the first hearts, contrast agent with 300 mg/ml iodine concentration (Iopromide, Utravist, Bayer Healthcare, Berlin, Germany) with a 60/40% contrast-to-saline ratio, was injected at an injection rate of 3 ml/s, with a total volume of 15 ml. As the fail rate in the first six porcine hearts was unexpectedly high (two completed and four failed porcine hearts), and one of the hypotheses was that coagulability due to iodine contrast could possibly affect the state and rhythm of the heart, there was a switch to an ionic low osmolar iodine contrast with anti-coagulating effect in the last four porcine hearts [18]. In the last four hearts (three completed the entire imaging protocol), a contrast agent with 320 mg/ml iodine concentration (Hexabrix, Guerbet, Paris, France) was injected into the blood stream with a 55/45% contrast-to-saline ratio at an injection rate of 3 ml/s, with a total volume of 15 ml. Dynamic scans started 5 s prior to contrast injection to have multiple baseline measurements, with a total scan time of 60 s. Dynamic scans were performed in shuttle mode during end-systole (Online Movie 1). The scanner shuttled between two table positions while acquiring information of the inflow of contrast in the myocardium. The images were combined to form one image and were acquired once every two or three heartbeats. The blood pool was refreshed after each set of stenosis grade scans, to prevent the build-up of contrast in the circulating blood pool of approximately 5 l.

Total heart coverage of the shuttle mode was 73 mm: twice the detector width of 38.4 mm, with an overlap of 10%. The combination of high heart rate and shuttle mode resulted in dynamic scanning every second or third heart beat (every 2–3 s). Acquisition parameters were: tube voltage 100 kV, 350 mAs per rotation and a gantry rotation time of 285 ms. The heart and inflow tube were placed in the FOV, which had a matrix size of 512 × 512. The FOV included the short-axis view of the heart. A part of the inflow tube to the heart was looped through the FOV to allow calculation of an input function as reference for the perfusion of individual myocardial segments.

### Image reconstruction and analysis

**Fig. 3:**
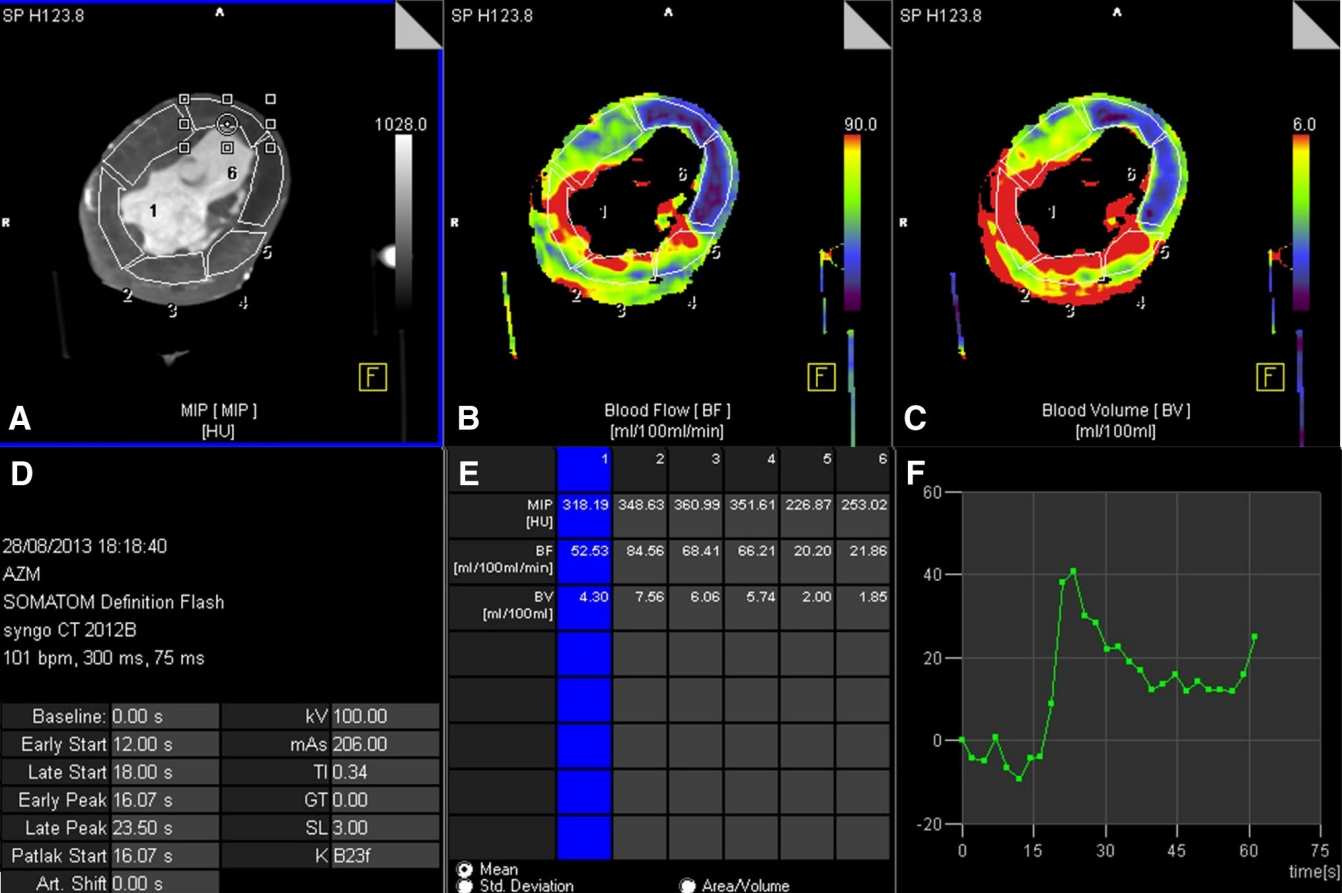
Syngo.via blood flow analysis. Perfusion analysis in Syngo VPCT myocardium for an induced stenosis with an FFR of 0.30. **a**–**c** Show MIP of the HU, a color map of the myocardial blood flow, and a color map of myocardial blood volume, respectively. **d** shows scan parameters and timing parameters derived from the HU curve. In **e** the measurements for the six separate segments are displayed and **f** shows the HU curve of segment 6

CT myocardial perfusion datasets were reconstructed in short-axis with 3.0 mm slice thickness, 1.5 mm increment, and smooth, quantitative, B23f reconstruction kernel, with weighted filtered back projection, including an iodine beam hardening correction algorithm. No additional reconstruction software was used. Short axis datasets were analysed using commercially available software, Volume Perfusion CT (VPCT) Myocardium (MMWP VA41A, Siemens Healthcare GmbH, Forchheim, Germany). An adapted AHA 17-segment model, with exclusion of the apex, was used to broadly define the myocardial segments [19]. This model was used to provide the reader with an indication on the location of the myocardial segments. Based on the total occlusion scan, we were able to determine the exact vascular territory of the CX artery. Drawing of the contours was finalized based on the myocardial area supplied by the CX artery as judged from the total occlusion scan. The background contrast build-up was monitored by analysing the HU values in the inflow tube. An example of the measurements is shown in Fig. 3. The apex was excluded as this segment was often not part of the scan range. The perfusion pattern based on the occlusion scan was used to determine which myocardial segments were perfused by the Cx. Myocardial segments in the Cx territory were defined as ischemic segments for all dynamic perfusion scans at all stenosis grades, while the other segments were defined as non-ischemic segments. The VPCT software calculated blood flow parameters for every tissue voxel using the arterial input function (AIF) and the tissue attenuation curve (TAC) of the myocardial segments [20]. Mean values of voxels within manually drawn segments were used to calculate mean myocardial blood flow (MBF_CT_) in ml/100 ml/min per segment, both for ischemic segments and non-ischemic segments. Mean MBF per myocardial segment was determined by averaging the MBF values of the three dynamic perfusion scans and was calculated per segment for each stenosis grade for each heart. Mean MBF was determined in 400 (5 hearts × 5 experiments × 16 segments) myocardial segments of which 115 were classified as ‘ischemic’. Mean MBF values over three dynamic perfusion scans per stenosis grade per heart were used to determine differences between ischemic and non-ischemic segments.

### Microsphere injection protocol

Fluorescent microspheres were injected in the three of the five hearts at several stenosis grades: no stenosis, FFR 0.70, FFR 0.50 and FFR 0.30, after each set of three dynamic CT scans (Fig. 2). Microspheres (300,000 spheres; Dye-Trak, Triton Technology, San Diego, California) with different coloured labels were used, each representing a different stenosis grade: yellow, lemon, orange and persimmon. These microspheres were infused during a 30 s interval. At the same time, the ABF to the heart was monitored. Microspheres had a diameter of 15 µm, and lodged in the microcirculation. After completion of the experiment, the hearts were sliced into pieces of approximately 5 g and these samples were dissolved in alcoholic-KOH, leaving only fluorescent microspheres of which the fluorescence was determined using a previously described method [21]. Absolute myocardial blood flow (MBF_MIC_) in ml/g/min per sample was calculated from the total blood flow in the setup (measured by the aortic flow probe) and the ratio of fluorescence in the sample and total fluorescence in all samples.

MBF_CT_ measurements were related to reference fluorescence measurements of MBF_MIC_ per minute. Ischemic segments were defined based on the total occlusion scan results. The MBF _CT_ (ml/100 ml/min) was corrected to ml/g/min by dividing by 105 (the density of cardiac muscle is approximately 1.05 g/ml) [22]. Mean MBF_MIC_ was calculated per slice, resulting in six MBF measurements: basal, mid-ventricular and apical MBF_MIC_ for both ischemic segments and non-ischemic segments. These measurements were then correlated to the mean MBF_CT_ in the corresponding slices and segments.

### Statistical analysis

Statistical analysis was performed using SPSS 23 (IBM Corp, Armonk, NY). Pearson’s correlation was determined between stenosis grade and ABF and between stenosis grade and MAP. Independent variance tests were carried out to analyse whether the ischemic and non-ischemic segment groups showed normal distribution of perfusion measurements. Median increase in HU contrast value was determined between the three repeated measurements for each stenosis grade to monitor contrast build-up before blood pool refresh. Thereafter, the Mann–Whitney *U* test for equality was performed. Median values and interquartile range (IQR) of mean MBF values were compared between non-ischemic segments and ischemic segments, at each FFR occlusion grade. Each perfusion scan was treated as a separate perfusion measurement, while the stenosis was induced for each myocardial perfusion scan separately.

Correlation between MBF_CT_ and MBF_MIC_ was determined analysing the fit of the linear regression models using Pearson’s correlation for separate hearts, and mean MBF values of three CT measurements at FFR 0.70, 0.50 and 0.30. Paired samples *T* tests were used to analyse general difference between MBF_CT_ and MBF_MIC_.

## Results

### Experiment success rate and model stability

All ten hearts successfully recovered stable sinus rhythm after warming up. In five out of ten porcine hearts, all perfusion scans at all stenosis grades could be completed. In the hearts with premature failure, aortic pressure started to increase while total aortic blood flow decreased, indicating development of myocardial oedema. One heart failed before scans were made, two hearts failed before applying a stenosis, and two hearts failed during the first two stenosis grades. CT perfusion analysis was performed only in the five hearts with successfully completed experiment.

**Fig. 4:**
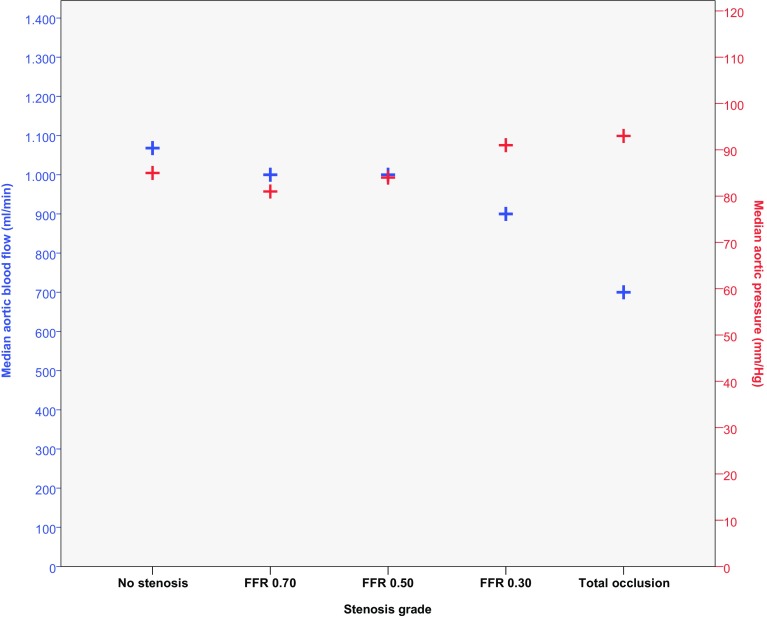
Aortic blood flow and blood pressure. Median aortic flow (*blue*) shows a decrease at increasing stenosis grades, while median aortic pressure (*red*) shows an increase towards higher stenosis grades

During the experiment mean heart rate ranged from 75 to 134 beats per minute. Overall, ABF ranged from 0.5 to 1.4 l min, with higher ABF at lower stenosis grades and in normal perfusion (Pearson’s correlation, R = −0.65, p < 0.0001). The opposite is shown for MAP, which increased at higher stenosis grades and was lowest for normal perfusion (Pearson’s correlation, R = 0.27, p < 0.05) (Fig. 4). An increase in HU was found for each set of repeated measurements before refreshing the bloodpool, median increase was 18.4 HU with 25 and 75 percentiles of 11.1 and 41.2 HU.

### Imaging results

**Fig. 5:**
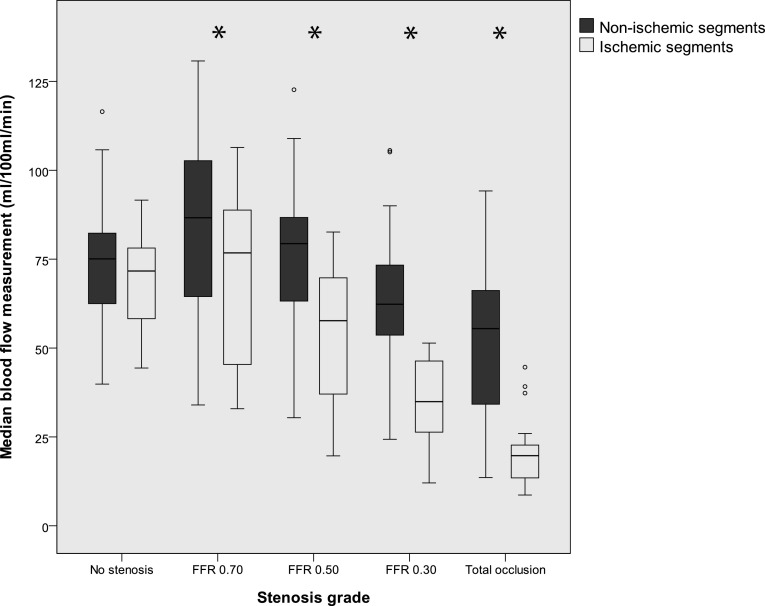
Myocardial blood flow at multiple stenosis grades. A significant difference in blood flow measurements between normal segments and segments with perfusion defect is found for stenosis grades FFR ≤ 0.70. *Indicative of statistical significance (p < 0.05)

The decrease in ABF shown in the standard setup measurements of aortic flow during the experiment (Fig. 4) is also visible in the MBF_CT_ in normal segments (Fig. 5).

There was a significant difference in MBF_CT_ between non-ischemic and ischemic segments for all stenosis grades with an FFR ≤ 0.70 (p < 0.05) (Fig. 5). Median MBF in non-ischemic segments ranged from 86.7 (IQR 63–102) ml/100 ml/min at FFR 0.70–55.5 (IQR 34–67) ml/100 ml/min at total occlusion, while median MBF in ischemic segments ranged from 76.8 (IQR 40–89) ml/100 ml/min at FFR 0.70–19.7 (IQR 12–23) ml/100 ml/min at total occlusion. A significant difference between non-ischemic and ischemic segments was found at an FFR of 0.70, with a median MBF of 86.6 and 76.7 ml/100 ml/min, respectively (p < 0.05). Mean standard deviation of the MBF_CT_ for the three CT measurements, per segment, was 16.1 ml/100 ml/min.

**Fig. 6:**
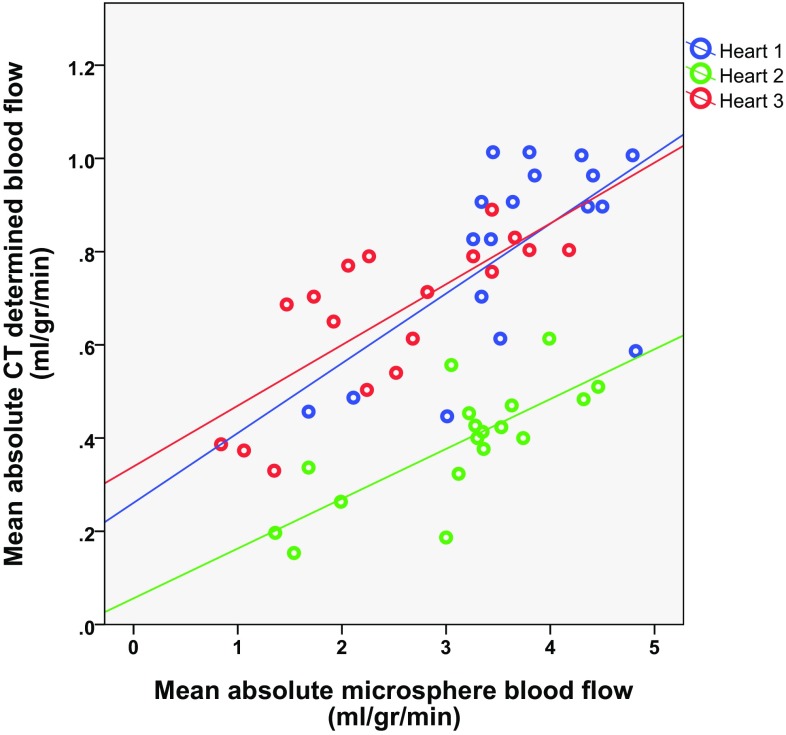
Correlation between CT-determined and microsphere-determined blood flow. For each heart a significant correlation was found between MBF_CT_ and microsphere-derived MBF. The results of separate hearts are displayed in *different colors*, along with the corresponding R^2^ of the linear fit for each heart

MBF_MIC_ and corrected MBF_CT_ showed significant correlation (Pearson correlation ranging from 0.62 to 0.76, p < 0.01) at induced stenosis grades of FFR 0.70–0.30 (Fig. 6). Overall, MBF_MIC_ was higher in comparison to MBF_CT_ with values ranging from 1 to 6 ml/g/min, where MBF values for CT ranged from 0.5 to 1.2 ml/g/min (p < 0.05).

## Discussion

In this study we intended to validate the quantification of myocardial perfusion in dynamic CT on second generation DSCT, using a controllable ex-vivo porcine heart model. The current study yielded several important findings. Firstly, results show high potential for quantitative dynamic CT perfusion analysis to distinguish between ischemic and non-ischemic myocardial segments. Secondly, quantitative CT measurements of MBF showed a considerable underestimation compared to microsphere-based MBF quantification. However, a clear positive correlation was found between CT-determined and microsphere-based MBF.

In the one heart that was analysed in the previous ex-vivo publication, no significant differences were found between non-ischemic and ischemic segments in case of no stenosis or FFR 0.70 [16]. In the segmental analysis of the five porcine hearts in the current study, we found a significant difference between segments with normal perfusion and segments with reduced perfusion for all stenosis with an FFR ≤ 0.70 grades. This implies that quantitative CT perfusion analysis can distinguish segments with and without a perfusion defect. The flow measurements in total occlusion scans were low, but not zero. This could be caused by collateral flow and leakage into the vascular territory supplied by an occluded artery in these experiments.

CT-determined and microsphere-determined MBF quantification showed moderate correlation, which indicates that high MBF_CT_ corresponds to high MBF_MIC_ and vice versa. However, in general, MBF_CT_ values for both non-ischemic and ischemic segments were significantly lower than MBF_MIC_ values. The MBF_MIC_ values had a large range. Still, MBF_MIC_ values were closer to the gold standard positron emission tomography measurements where cut-off values of 2.5 ml/g/min for ^15^O and 1.4 ml/g/min for ^82^Rb have been suggested to differentiate between ischemic and non-ischemic segments [23–25]. The MBF_CT_ values are not close to ‘real’ absolute myocardial blood flow. Ishida et al. already reported that the MBF_CT_ values determined by shuttle mode CT in patients are not representing real myocardial blood flow [26]. One of the hypotheses is that the temporal sampling of the shuttle mode scans is not sufficient to accurately calculate the first-pass MBF. Furthermore, Ishida et al. stated that the model used in the vendor-provided software is not calculating MBF but Ktrans, a volume transfer constant according to the Patlak equation model [26]. These results suggest that one can use visual analysis of dynamic CT MPI maps in the detection of myocardial perfusion defects. However, one should be careful with the interpretation of the quantified absolute MBF values.

The results in this study are based on experiments using the PhysioHeart^®^ setup, which allows direct control over ABF and MAP, and enables direct manipulation of the degree of stenosis in explanted porcine hearts. The ABF decreased while the MAP increased with increasing stenosis grades. This was expected because with higher stenosis grade, peripheral resistance increased, leading to higher MAP and lower ABF. Throughout the experiment ABF and MAP were kept as stable as possible. Stenosis grade, aortic pressure and flow were adjustable during the experiment, with the isolated heart setup being free of hormonal influences. The model is less complex as compared to in-vivo animal studies, providing more control and possibly better reproducibility. In this model the heart is already in stress state because of the excision and recovery from the cold-ischemic period during transportation so there is no need for pharmalogically induced stress [27]. The retrograde perfusion model used in this experiment was originally developed by Oskar Langendorff in 1895 [28]. In a review in 2007, Skrzypiec-Spring et al. concluded that the Langendorff model could be used to study ischemia [29]. Schuster et al. already showed potential for perfusion analysis using the Langendorff model in a clinical MRI scanner and concluded that this technique is feasible to use in MRI [30, 31]. In an earlier study, the Langendorff model was applied in CT, and feasibility of this technique in CT was shown in one porcine heart [16]. In in-vivo experiments by Bamberg et al. and Rossi et al., dynamic CT MPI analysis was analyzed at several stenosis grades. These studies showed that CT MPI was able to detect significant stenosis using dynamic perfusion analysis. In our study dynamic CT MPI differences in MBF were found between ischemic and non-ischemic segments, comparable to the results found in the in-vivo studies [7, 9]. Furthermore, more recent studies by Hubbard et al. and Fahmi et al., showed high potential for other CT modalities (Multidetector CT and Spectral CT) in the ability to evaluate myocardial blood supply [32, 33]. In addition to these studies we show that dynamic CT perfusion imaging using Dual Source CT has the ability to distinguish ischemic from non-ischemic segments based on MBF measurements at several FFR grades. A study by van der Pals et al. showed that T1–weighted MRI yielded comparable results of the area at risk in a canine model of myocardial infarction and reperfusion, compared to CT perfusion [34]. In our study, we focused on ischemia and quantification of myocardial blood flow instead of myocardial infarction. In our study, we did not perform MRI perfusion quantification because this porcine heart model was not MRI compatible.

This study has a number of disadvantages. Only 50% of the porcine heart experiments were completed. In five of the porcine hearts, the experiment ended prematurely. One of the hypotheses is that this was caused by development of oedema in the cardiac muscle. One of the factors that could prevent this is the anti-coagulating effect of ionic low osmolar iodine contrast. This was not specifically tested in this study, but we hypothesized this could be one of the factors affecting the state and rhythm of the heart, as in the first part of experiments, in which non-ionic iodine contrast was used, four of the six hearts stopped prematurely. For this reason, we changed to a different iodine contrast, which increased the success rate in the second part of the experiments (three out of four). Another reason for the low success rate could be the location of the stenosis. The Cx supplies a large part of the left ventricle and perfusion defects could result in arrhythmias and development of oedema. It is important to study these influencing factors to increase the success rate of the ex-vivo experiments in future experiments. The modified coronary integrity, contrast protocol and left ventricular pressure differ from the in-vivo situation. Furthermore, the baseline normal perfusion in the Cx territory already showed a decrease compared to the normal perfusion, as described above. Baseline FFR measurements in non-stenotic coronary arteries were not available, but could have ranged between 0.85 and 1.00 based on the setup. Furthermore, a particular difference with the in-vivo studies is that in our Langendorff model, retrograde flow was applied instead of normal antegrade flow. The arterial input function used in normal perfusion analysis is derived from blood that flows from the heart to distant organs, whereas in the current arterial input function, all blood traversed to the heart, in retrograde flow. So in in-vivo experiments, the arterial input function is based on blood that does not flow through the heart, whereas in our experiments all blood and contrast included in the arterial input function flows through the heart. This could be an additional reason for offset in the MBF_CT_ parameters using standard CT perfusion quantification models in the Langendorff mode. This experiment was performed in only a small number of porcine hearts. However, the analysis was based on differences between myocardial segments, which increased the power of the study. The analysis of perfusion was performed using manual segmentation which could have induced variability. Automatic software has not been developed for use in ex-vivo imaging, because landmarks are different compared to in-vivo imaging. Therefore, in ex-vivo experiments, manual delineation of automatic software cannot be used ex-vivo. Additionally, there is no material surrounding the heart. Therefore, the absolute HU values may differ from the in-vivo situation. However differentiation between normal and ischemic segments relies on relative measures of MBF based on tissue attenuation curves, and quantification of MBF should not be influenced by a potential baseline shift in HU values. Further research should be performed to analyse whether it is possible to link the CT perfusion blood flow measurements to real-time absolute flow measurements in the heart. Another issue to study is the reason for the underestimation of MBF_CT_ compared to absolute MBF according to microsphere measurements and how this can be solved.

## Conclusion

These studies in an ex-vivo porcine Langendorff perfused heart model demonstrate that CT MPI can be used to determine regional differences in myocardial perfusion parameters, based on severity of coronary stenosis. Significant differences in MBF could be measured between non-ischemic and ischemic segments.

## Electronic supplementary material

Below is the link to the electronic supplementary material.

Supplementary material 1 (WMV 2271 KB)
